# The Use of Surrogate Data in Demographic Population Viability Analysis: A Case Study of California Sea Lions

**DOI:** 10.1371/journal.pone.0139158

**Published:** 2015-09-28

**Authors:** Claudia J. Hernández-Camacho, Victoria. J. Bakker, David Aurioles-Gamboa, Jeff Laake, Leah R. Gerber

**Affiliations:** 1 School of Life Sciences, Arizona State University, Tempe, Arizona, United States of America; 2 Department of Ecology, Montana State University, Bozeman, Montana, United States of America; 3 Laboratorio de Ecología de Pinnípedos ‘‘Burney J. Le Boeuf”, Centro Interdisciplinario de Ciencias Marinas, Instituto Politécnico Nacional, La Paz, Baja California Sur, México; 4 National Marine Mammal Laboratory, Alaska Fisheries Science Center, National Oceanic and Atmospheric Administration, Seattle, Washington, United States of America; University of Missouri Kansas City, UNITED STATES

## Abstract

Reliable data necessary to parameterize population models are seldom available for imperiled species. As an alternative, data from populations of the same species or from ecologically similar species have been used to construct models. In this study, we evaluated the use of demographic data collected at one California sea lion colony (Los Islotes) to predict the population dynamics of the same species from two other colonies (San Jorge and Granito) in the Gulf of California, Mexico, for which demographic data are lacking. To do so, we developed a stochastic demographic age-structured matrix model and conducted a population viability analysis for each colony. For the Los Islotes colony we used site-specific pup, juvenile, and adult survival probabilities, as well as birth rates for older females. For the other colonies, we used site-specific pup and juvenile survival probabilities, but used surrogate data from Los Islotes for adult survival probabilities and birth rates. We assessed these models by comparing simulated retrospective population trajectories to observed population trends based on count data. The projected population trajectories approximated the observed trends when surrogate data were used for one colony but failed to match for a second colony. Our results indicate that species-specific and even region-specific surrogate data may lead to erroneous conservation decisions. These results highlight the importance of using population-specific demographic data in assessing extinction risk. When vital rates are not available and immediate management actions must be taken, in particular for imperiled species, we recommend the use of surrogate data only when the populations appear to have similar population trends.

## Introduction

Population viability analysis (PVA) is a quantitative tool to predict population trends and extinction probabilities for imperiled species [1]. PVA models contribute to the development of effective management plans and have become a powerful tool for conservation biologists and other practitioners [2–4]. Reliable data necessary to parameterize demographic PVAs are seldom available for long-lived individuals because long-term monitoring is often impossible for both logistical and financial reasons [5, 6]. This is particularly relevant for small and endangered populations due to difficulty observing individuals, the inaccessibility of study areas, and legal and ethical limits on data collection techniques.

When data are lacking, surrogate data are commonly used to parameterize PVA models [6, 7]. Surrogate data have been used for viability assessments for a wide variety of taxa, including insects [8], birds [9, 10], reptiles [11], and mammals [12, 13]. The scope of data borrowing ranges from the use of data from nearby populations of conspecifics to the use of data from distant populations of related species.

The implicit assumptions when using surrogate data are that the target population or species has similar life history traits and experiences similar ecological conditions to the surrogate and thus their demographic rates are similar. While life history traits might show general concordance among different populations of the same or closely related species [14], age- and sex-specific vital rates oscillate within a range of values under different environmental and demographic conditions due to environmental stochasticity, density dependence in vital rates, and natural and anthropogenic disturbances [6, 7, 15]. Thus, it may often be unrealistic to assume that the demography of one population can serve as a proxy for that of another, particularly if their population trends differ (growing vs. declining).

Some studies have shown that surrogate data may provide useful insights regarding the conservation of an ecologically similar species [e.g., 10]. However, several recent studies have reported that populations of the same species can have significantly different demographic rates [16, 17], which can affect the predictions of PVAs that use surrogate data [7].

In most situations where surrogate data are used, it is impossible to assess the reliability of resulting models because data on the target species are absent. In this study, however, we were able to evaluate the reliability of the surrogate data using a series of stochastic demographic PVAs for California sea lion (*Zalophus californianus*) colonies in the Gulf of California, Mexico, by comparing predicted retrospective population dynamics to long-term population count records for each colony. These records indicate that the sea lion population in the Gulf has decreased ~20% over the last two decades, with colonies in the upper and central Gulf undergoing the most marked population declines [18]. Considering this overall population decrease it is important to re-assess the conservation status of individual colonies in order to estimate extinction risk and inform colony-specific management actions in the Gulf. Thus, we sought to examine the extent to which data from one well-studied breeding colony (Los Islotes) could be used to create realistic demographic matrix models for two other colonies that have not been studied as extensively (San Jorge and Granito). We compared retrospective model predictions to a 28–30 year count record for each colony. Finally, we examined any mismatches between observed count trends and those predicted using surrogate demographic data in order to identify the life stages most likely to be responsible for the population decline.

## Materials and Methods

### General approach

**Fig 1:**
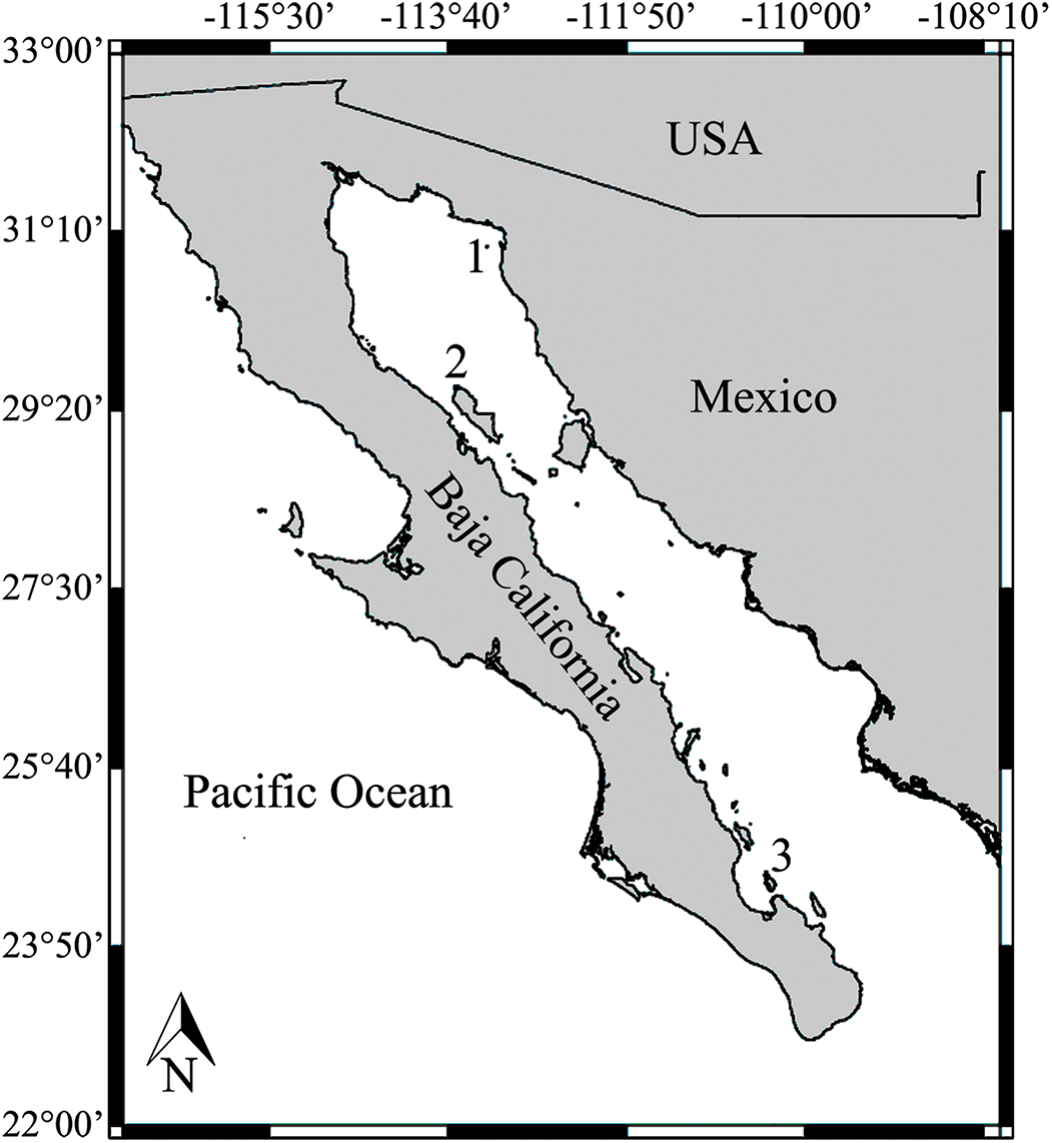
Map of the study area: 1. San Jorge (SJ), 2. Granito (G), and 3. Los Islotes (LI).

The Los Islotes (LI) sea lion colony has been monitored intensively during the last three decades (Fig 1). A long-term population count series is available for this population [18–20], with counts made systematically during the breeding season when sea lions of all age- and sex-classes congregate at reproductive colonies. In addition, a sample of permanently marked adults has enabled the estimation of adult age- and sex-specific survival rates and old adult age-specific birth rates. In contrast, the colonies at San Jorge (SJ) and Granito (G) have been counted during the same period of time (although not with the same regularity as LI), but lack a permanently marked sample and thus adult age- and sex-specific vital rates have not been estimated. The three breeding colonies differ in size, population trajectories, and potential anthropogenic threats (S1 Table). In this study, pup and juvenile survival rates were estimated for all three colonies using temporary marking, giving us colony-specific data for nearly all LI demographic rates (except prime-age female birth rates which were taken from females from California, [21]), but only pup and juvenile survival rates for G and SJ. We developed stochastic demographic PVA for each colony using the respective pup and juvenile survival probabilities. For SJ and G, we used surrogate data for adult survival and female birth rates. Starting in 1981 (the first year systematic counts were made), we estimated population trajectories for each colony using both demographic and count-based PVA models. We evaluated the fit between the observed data and the model predictions for all three colonies; when models failed to predict the observed population trajectory based on the count data, we identified the adult survival rates that would result in more accurate predictions.

### Survival estimates for younger age classes

Although it was not logistically feasible to permanently mark adults, we were able to mark and monitor sea lion pups to obtain colony-specific demographic data for younger age classes. Specifically, we estimated pup and juvenile survival rates for the SJ, G, and LI breeding colonies based on the resighting histories of individuals tagged as pups between 2004 and 2007 using a mark-recapture model.

We visited each colony during the breeding season (July and August) to tag sea lion pups (S2 Table). Pups were temporarily marked with plastic flipper tags (Dalton I.D. Systems, U.K., Long-Term Jumbo Tags) on both front flippers [22]. All field protocols were approved by the Animal Care and Use Committee at Arizona State University. This study was conducted under the following SEMARNAT (Secretariat of Environment and Natural Resources) research permits: No. 240996-213-03, DOO 750.-4172/97, DOO 750.-4443/98, NUM/SGPA/DGVS 04311, 04160, 05325, and 03269.

Two pairs of observers recorded the presence of tagged sea lions at each colony during two or three 6–8 day periods during the summer breeding season (S1 File). We also visited each colony two or three times (except LI, which was visited only during winter) during the spring, fall, and/or winter (S2 Table).

We built a release resighting history for each tagged sea lion from the four cohorts using the four annual marking events and the resighting data collected on each colony from January/February 2005 to February 2008 (Table A in S1 File). We classified individuals into two age classes: pups (< 1 year old) and juveniles (1–4 years old) [20]. We used the RMark interface [23] to develop a sequence of Cormack-Jolly-Seber models [24] using MARK [25] to estimate apparent survival (φ) and resighting probability (*p*). We defined 75 models for φ and eight models for *p* with different combinations of age class, sex, time, colony, and other covariates (i.e., pup weight, female behavior, female and pup density in territorial areas, and entanglement rates) and their interactions (models proposed and descriptions of variables and methods are summarized in Table A and Table B in S2 File, S3 File).

We selected the best models using the small sample Akaike information criterion (AIC_c_) [26]. The best models have the lowest AIC_c_, ∆AIC_c_ < 2, and the greatest weights (ω). The relative importance of each variable was computed by summing the Akaike weights across the best models in which a specific variable occurs [26]. We calculated model average estimates, standard errors, and confidence intervals for the parameters including models with ∆ AIC_c_ < 4. All *p* models tested were inferior, with a ∆AIC_c_ ≥ 20, except the full interaction model: *p* (sex*age*colony*time). Thus, we fit each φ model to the full interaction model to obtain the 75 candidate models. We used RELEASE to compute goodness of fit test and estimate overdispersion ($\hat{c}$) for the global model (φ_colony*age*time*sex_ *p*_colony*age*time*sex_) by splitting the data into 24 groups (four female and four male cohorts for each colony).

### PVA

**Fig 2:**
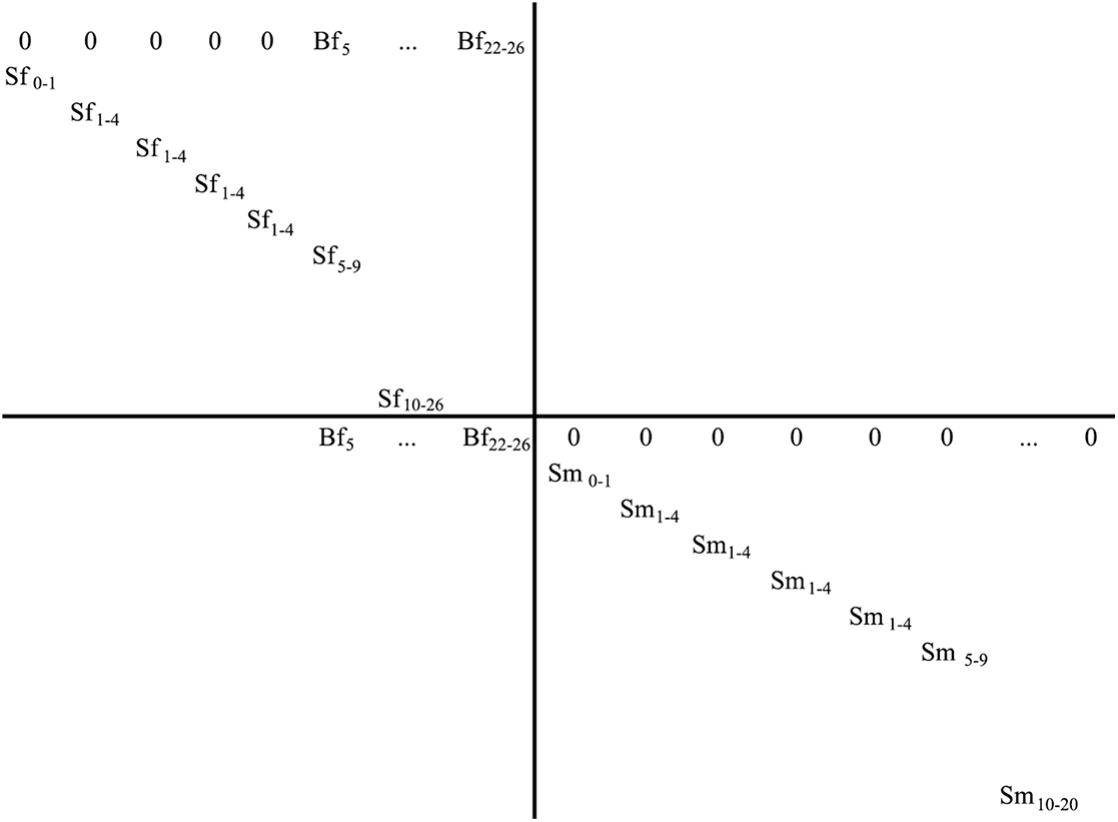
Schematic representation of the age- and sex-structured matrix used to develop the demographic PVA model. *Bfi* = age-class-specific fertility, *Sfi* = female survival rate at age class *i*, and *Smi* = male survival rate at age class *i*.

We built age- and sex-structured stochastic demographic matrix models (52 x 52) to predict population change through time (λ) at SJ, G, and LI (Fig 2). The top half of the matrix describes the dynamics for females, the bottom half for males. The top row represents age-class specific fertility (i.e., Bfi = birth rate at age-class i * female survival rate at age-class i [Sfi]) while the diagonal values represent age-class specific survival rates (Sfi and Smi). The matrix assumes a post-breeding count and imposes a maximum lifespan of 26 years for females and 20 years for males. Colony-specific survival rates derived from the mark-recapture analysis were used for male and female pups and juveniles. Survival rates for prime-age adults (5–9 years old) and old adults (≥ 10 years old) of each sex come from a previous mark-recapture analyses done for five cohorts of sea lions branded as pups and resighted at LI over 26 years (1981–2006, [20]). There was no support for temporal variation in survival rates over this time period. Age-specific birth rates for prime-age females (5–9 years old) were obtained from San Miguel Island (SMI), California and are thus surrogate data [21], while that for old adult females (≥ 10 years old) come from a 12 year study at LI colony (1994–2006, [19]).

Annual survival rates for pups and juveniles were obtained from the best survival model that included year effects (i.e., φ [14]) (Table 1). This model, which had a ∆AIC_c_ < 2 compared to the best supported model, includes island, age, sex, and year effects, and allowed us to estimate temporal variance in survival rates. We estimated the process variance associated with stochastic environmental processes [27, 28] for birth rates (using the method described in [1, 28]) and survival rates (using the process variance option in MARK described in [29]) (Table A and Table B in S4 File), and modeled environmental stochasticity by sampling survival and birth rates for each time step of the simulation from beta distributions defined by the estimated means and process variances [1]. The beta distribution is a continuous distribution of probabilities confined to the interval 0–1 and can take a variety of forms; thus, it is ideal for modeling survival rates [1]. We enforced the observed correlation structure between age- and sex-specific survival rates and birth rates by generating correlated random matrices for each annual increment [1]. Because LI was the only colony for which we could estimate a full correlation matrix, we applied these correlations for LI to all colonies. Thus, correlations were an additional form of surrogate data for G and SJ. To test the importance of correlations to our results, we ran simulations without any correlations among demographic rates and the results were qualitatively the same; thus correlations based on surrogate data were not considered a source of bias in our projected population trajectories.

**Table 1:** Model selection results for the 15 best supported Cormack-Jolly-Seber models. In all cases, the *p* model was: *p*(1) = *p*_island*age*time*sex_. Φ and p represent age-specific survival and recapture probability respectively.

Model	N_par_	AIC_c_	∆AIC_c_	Weight (*ω*)	Deviance
φ (colony + sex)	112	5219.501	0.000	0.153	870.612
φ (colony + sex + age)	113	5221.032	1.531	0.071	869.906
φ (colony + time + sex + age)	117	5221.296	1.795	0.062	861.199
φ (sex + pup*density)	112	5221.849	2.349	0.047	872.961
φ (colony + time + sex)	116	5222.027	2.526	0.043	864.176
φ (sex+ pup*density + juvenile*time)	115	5222.618	3.118	0.032	867.012
φ (sex + pup*aggression + pup*density)	113	5222.791	3.290	0.030	871.665
φ (sex + pup*density + pup*weight)	113	5222.933	3.433	0.028	4983.795
φ (sex + pup*density + pup*density^2^)	113	5223.019	3.519	0.026	871.894
φ (colony)	111	5223.036	3.536	0.026	876.382
φ (sex)	110	5223.190	3.689	0.024	878.769
φ (colony + time + age)	116	5223.320	3.819	0.023	865.469
φ (colony + age)	112	5223.414	3.914	0.022	874.526
φ (colony + pup*time + sex)	116	5223.456	3.955	0.021	865.605
φ (colony + sex*age)	115	5223.456	3.956	0.021	867.85

We simulated a total of 5000 replicate retrospective population trajectories for each colony to characterize stochastic population dynamics. To permit comparison of count data with the population trajectories predicted by the PVA model, we initiated each simulation at the total number of sea lions present in 1981 (the year systematic counts began) and ran them through 2008. Due to financial constraints, it was not possible to visit all three colonies every year during the study; thus, for some years we are missing count data, particularly for SJ. There were 24, 19, and 10 annual counts for LI, G, and SJ respectively. To obtain the starting vector of individuals in each sex- and age-class, we extrapolated the total population at the stable age distribution from the female counts (LI = 63, G = 746, and SJ = 2,034). Although our model used a two-sex matrix, only the data on females were used to initiate models and for the different comparisons because we assumed the proportion of adult females on the beach vs. at sea (~observation error) would be less variable between years than for animals of other age classes [30]; thus eliminating the greater uncertainty associated with the count data for other age classes. This assumption is based on the fact that: 1) female sea lions are permanent residents and are highly philopatric [20]; 2) counts were performed during the breeding season every year, reducing the variation associated with seasonal changes in the distribution of prey and the energy demands of pups [31]; and 3) the duration of feeding trips during that time of the year is the same for all three colonies and is determined by local environmental conditions around the islands [32, 33].

Models were projected for 28–30 years (1981–2008 for SJ and G, 1981–2010 for LI). The fit of the stochastic model was assessed by fitting exponential lines to the observed data, with the intercept fixed at the year counts were initiated (1981). To do so, we used linear least squares curve fitting, employing the nlinfit function in Matlab (version 8.1) to fit a line to the log of the count data. To further quantify the fit of the simulated models, we calculated the proportion of years in which the 90% confidence intervals (CI) of the simulation models included the best fit line for the observed counts. We also performed a count based PVA using a diffusion approximation model [34]. We used female count data from 1981 to 2008 for SJ and G, and from 1981 to 2010 for LI so that the estimates from the demographic and count based PVAs would be comparable.

For colonies where there was a strong mismatch between predictions from the demographic PVA and the observed data, we were interested in finding the female adult survival rate that would result in simulated trajectories that fit the observed population trend. To do so, we searched for a prime-age adult female survival rate that minimized the mean absolute error (MAE), a statistical measure that expresses average model prediction error [35] between the count data and the PVA simulated values. We assumed that old adult female survival was a constant proportion of prime-age adult survival.

Finally, we estimated and compared the stochastic growth rate (λ) and geometric mean population size [1, 34] for both the count and the simulated data from the demographic PVA.

## Results

### Survival

The most general model fit the data (χ^2^ = 76.78, df = 84, p = 0.3) and there was no evidence of overdispersion ($\hat{c}$ = 0.91). The top 15 models had considerable support although no single model was superior. Age, sex, colony, and time were recurrent variables in the top models (Table 1). All of these models included sex but only a few included other covariates (pup weight and female density). Colony and sex had higher relative variable importance (0.44 and 0.56, respectively) than the rest of the variables (< 0.2). Pup and juvenile survival rates differed between colonies, but were relatively high overall (Table 2). Females had lower survival rates than males in all age classes at all colonies (Table A and Table B in S4 File for further details on survival rates and resighting rates).

**Table 2:** Model average estimates of age-specific survival probabilities (φ) for male and female California sea lions at San Jorge (SJ), Granito (G), and Los Islotes (LI). Survival probability (φ) varies with time, age, sex, and colony. CI = Confidence interval.

		Males			Females		
Age class	Time	φ	SE	95% CI	φ	SE	95% CI
**Pups**							
**SJ**	2004	0.866	0.081	0.621, 0.962	0.722	0.109	0.474, 0.882
	2005	0.914	0.055	0.731, 0.977	0.812	0.080	0.607, 0.924
	2006	0.919	0.055	0.698, 0.983	0.820	0.097	0.557, 0.943
	2007	0.872	0.110	0.499, 0.979	0.752	0.151	0.383, 0.937
**G**	2004	0.974	0.025	0.846, 0.996	0.934	0.060	0.679, 0.990
	2005	0.981	0.020	0.861, 0.998	0.950	0.050	0.709, 0.993
	2006	0.981	0.022	0.840, 0.998	0.948	0.055	0.673, 0.994
	2007	0.968	0.034	0.776, 0.996	0.921	0.077	0.597, 0.989
**LI**	2004	0.938	0.044	0.772, 0.985	0.856	0.076	0.640, 0.952
	2005	0.938	0.030	0.835, 0.992	0.909	0.055	0.729, 0.974
	2006	0.965	0.032	0.809, 0.994	0.915	0.062	0.694, 0.981
	2007	0.921	0.060	0.696, 0.984	0.829	0.100	0.550, 0.950
**Juveniles**							
**SJ**	2005	0.875	0.088	0.591, 0.971	0.754	0.097	0.524, 0.896
	2006	0.906	0.072	0.647, 0.981	0.800	0.101	0.538, 0.932
**G**	2005	0.957	0.035	0.806, 0.992	0.901	0.066	0.681, 0.975
	2006	0.964	0.034	0.800, 0.994	0.911	0.069	0.658, 0.982
	2007	0.920	0.078	0.588, 0.989	0.839	0.118	0.486, 0.967
**LI**	2005	0.934	0.052	0.732, 0.987	0.860	0.072	0.656, 0.952
	2007	0.876	0.118	0.456, 0.984	0.778	0.149	0.392, 0.950

### PVA

**Fig 3:**
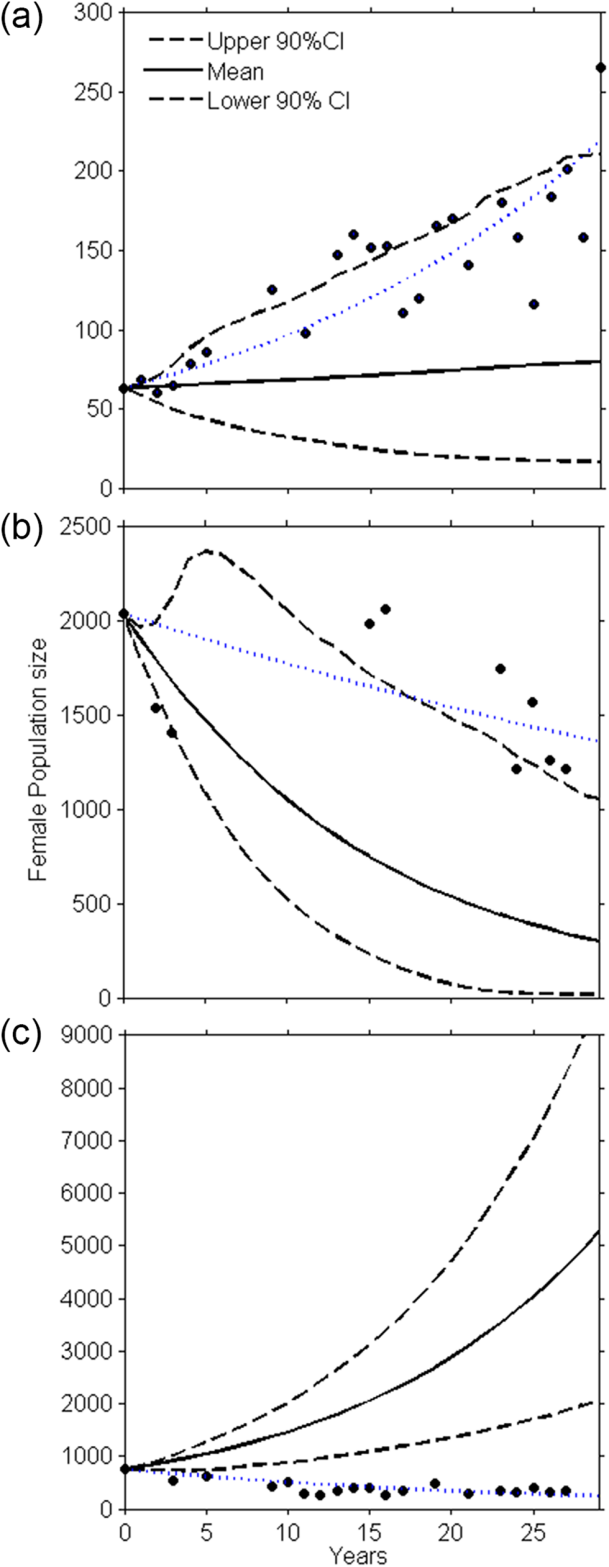
Mean predicted population projection (solid lines) and 90% upper and lower CI (black dotted lines) obtained from the demographic model. The blue dotted lines represent the best (exponential) fit to the observed counts. Dots represent counts, with year 0 equal to 1981. (a) Los Islotes, (b) San Jorge, and (c) Granito.

Population trajectories obtained from the stochastic demographic model predicted population increases for G and LI. The predicted retrospective population trends overlapped with the observed count trends when surrogate data were used, with the exception of one colony: G (Fig 3). For G, none of the years on the best fit line were included in the 90% CI of the simulation models; thus, there was a clear mismatch between the predictions (population increase) and the count data (population decrease). For SJ, both suggested a declining population and 0.61 of the years on the best fit line were included in the CI. At LI, where site-specific demographic data were used to parameterize the model (except for prime-age female birth rates), the average and upper CI population projections generally overlap with the observed population trend for most of the trajectory, with 0.97 of the years on the best fit line being included in the CI. The actual count data and model predictions for ending female population sizes vary considerably: 1,214 vs. 375 (90% CI 45–1,215) at SJ, 334 vs. 4,429 (90% CI 1,900–8,286) at G, and 266 vs. 84 (90% CI 14–214) at LI.

**Fig 4:**
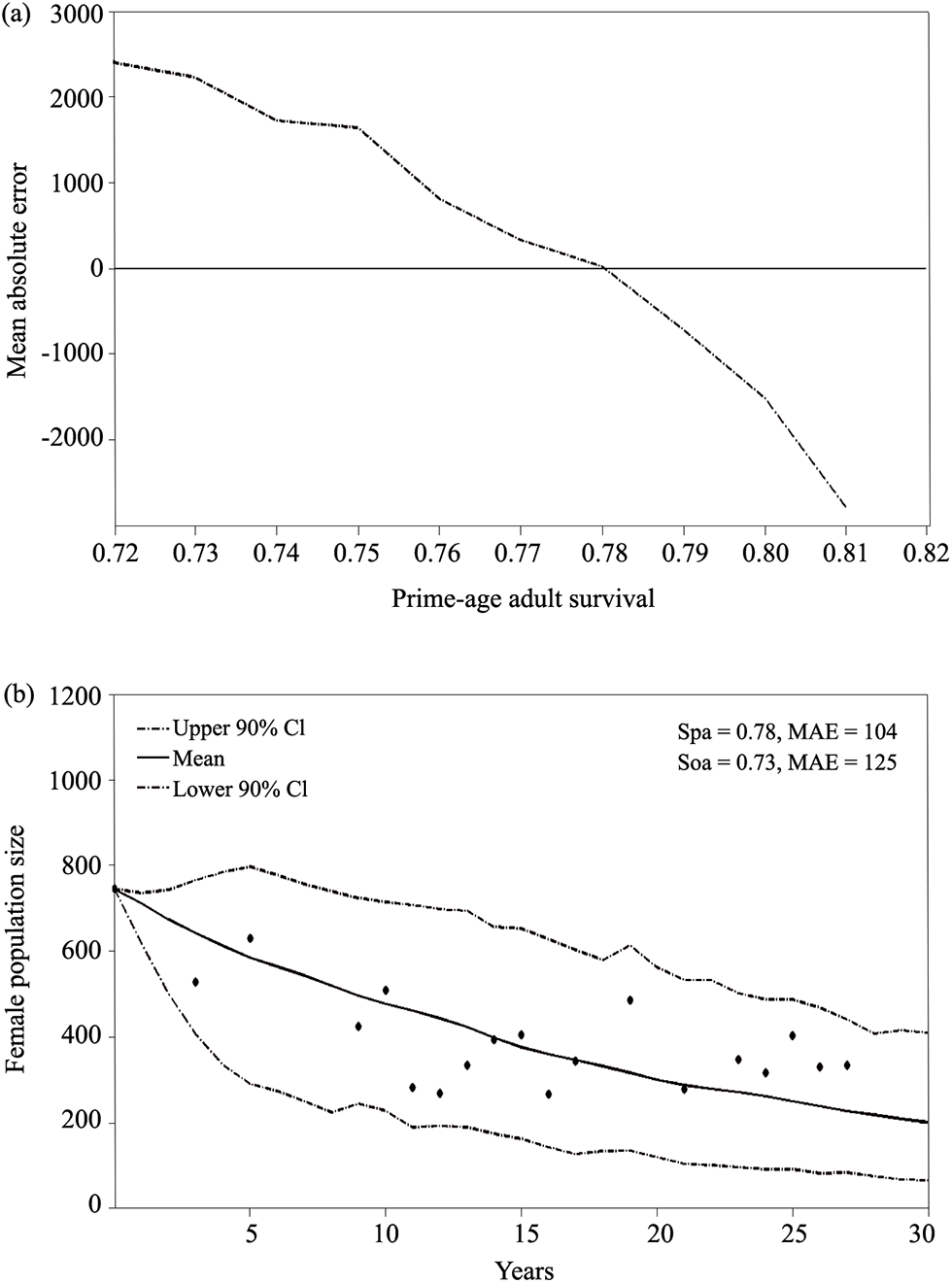
Mean absolute error (MAE) between the count data and the simulated population values based on a range of survival values (0.72–0.82) for prime-age adult survival at G (a). Survival values that minimized the MAE resulted in simulated trajectories that fit the observed population trend at G (b). *Spa* = prime-age adult survival, and *Soa* = old adult survival.

The survival rate that resulted in simulated trajectories that best fit the observed population trend for G was 0.78 for prime-age adult females and 0.73 for old adult females (Fig 4). These values are notably lower than the surrogate survival rates (0.97 and 0.91, respectively) from LI used in the original model.

**Fig 5:**
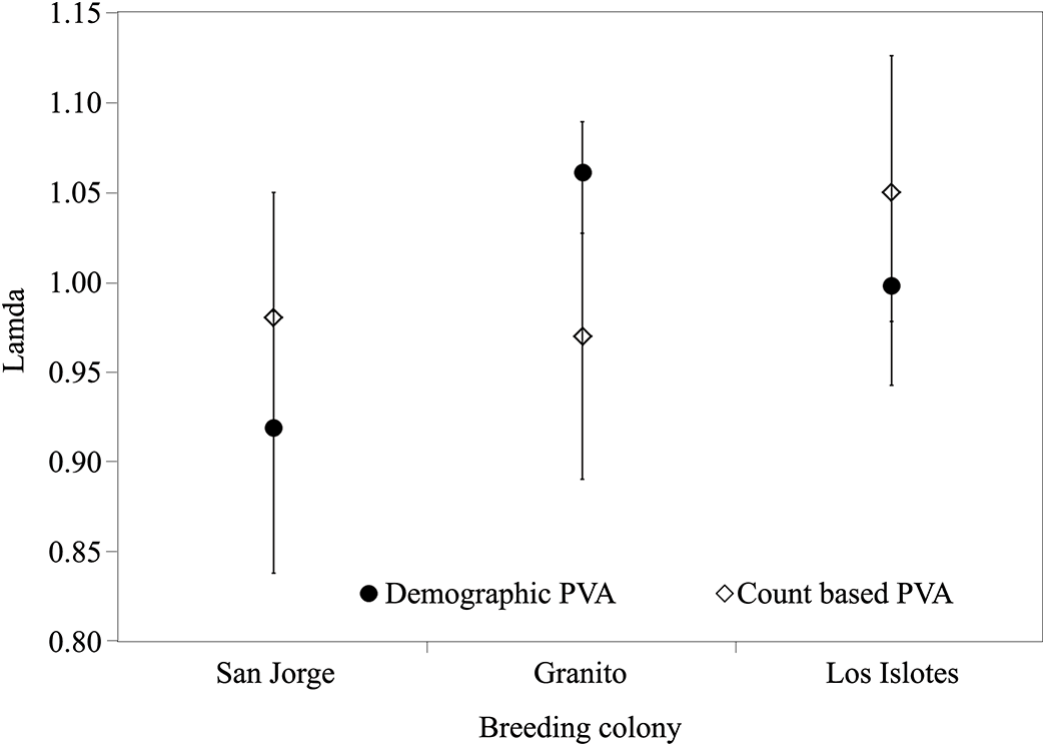
Stochastic growth rate values and 90% CI estimated from the female counts based PVA and stochastic demographic model.

The demographic and count-based PVAs for LI yielded similar stochastic λ values, suggesting a stable or increasing trend, although the demographic PVA results are more pessimistic (Fig 5). For SJ, both stochastic λ values suggested a declining population, with the demographic PVA again providing more pessimistic output. For G, stochastic λ values were markedly different: the stochastic λ value obtained from the demographic PVA was far more optimistic than that from the count-based PVA, resulting in disparate growth predictions (Fig 5).

## Discussion

Sea lion colonies in the Gulf of California showed distinct population trends and the survival rates of pups and juveniles differed, but discordance among observed population trajectories, estimated survival rates, and predicted population trends in the northern Gulf colonies calls for greater scrutiny by scientists and managers. Past studies have shown that the dynamics of each breeding colony vary with local environmental conditions (e.g., food availability) and other regional factors (e.g., degree of incidental bycatch) [18, 36]. In addition, colonies are spatially and genetically structured into three to four subpopulations (north, which includes SJ, northern midriff, which includes G, southern midriff, and south, which includes LI) exhibiting distinct ecological characteristics [37–40]. Thus, it is not surprising that survival rates for pups and juveniles differed among colonies. The relatively high pup and juvenile survival rates at G may translate to an increased growth rate in the future. However, this species’ population growth rate is most sensitive to juvenile and adult survival rates [36]. Thus, significant growth would only be expected if the survival rates of the adult age-classes also increased significantly, far surpassing the predictions of the simulated trajectories.

Annual variability in pup survival in all colonies (2004–2007) was lower than that observed at LI between 1980 and 1984, when a two-fold difference (0.55 vs. 0.998) was registered between the lowest and highest probabilities. In the present study, the biggest difference between the lowest (0.72) and highest (0.82) pup survival rates was identified for females from SJ. Unlike populations from California’s Channel Islands, variation in the size of the sea lion population in the Gulf of California is not associated with El Niño events [41], but rather appears to be related to local fluctuations in prey availability [42]. The population trends of some California sea lion rookeries in the central Gulf—including G—are correlated with Gulf sardine abundance, which was low between 2004 and 2007.

Only a few of the site-specific covariates were included in the top survival models. Noticeably absent were entanglement rates, which have apparently increased in the last two decades in the northern Gulf and may represent a threat to population viability [36, 43]. Young individuals are more susceptible to getting caught in fishing nets, and this mortality has contributed to the population decline of some pinnipeds (e.g., Northern fur seal, *Callorhinus ursinus*) [44]. In this study, evidence of entanglement was opportunistically recorded while carrying out the population counts. Thus, an unknown, and likely considerable, proportion of entangled individuals were missed. Increased effort should be made to locate entangled sea lions with systematic visits to colonies throughout the year to better assess the importance of this mortality factor.

Our PVA results demonstrate that the use of species- and region-specific surrogate data to predict population trends may yield misleading information for conservation managers. When fit with colony-specific demographic data for LI, our models yielded a reasonable fit to observed count data for LI and SJ. For LI, we attempted to use only site-specific demographic data and, although it would have been preferable to use birth rates exclusively from LI, we do not think the use of prime-age female birth rates from SMI biased our results because birth estimates for old adult females from LI were similar to those reported for females of the same ages at SMI [21]. One factor that may have biased our results is that prime-age (5–9 years old) and old adult (≥ 10 years old) survival rates come from a mark-recapture analysis of five cohorts of sea lions branded as pups and resighted at LI over 26 years (1981–2006); thus, current survival rates could be higher than those derived from the mark-recapture analysis resulting in lower predicted growth.

However, we found a dramatic mismatch between model predictions and count data for G. If conservation plans were based on the demographic PVA parameterized with surrogate data, managers would conclude that this colony is thriving when it is actually declining.

Female adult survival at G is a potential area of concern and an important driver of population growth. The adult female survival rates that resulted in simulated trajectories that fit the observed G population trend were notably lower than those observed at LI and for younger age classes on G itself. These results were surprising because it is unusual for adult female otariids, and long-lived mammals in general, to have lower survival estimates than younger age classes [20, 45–47]. Where food availability is limited, it is not uncommon for breeding females to be more affected than other ages and sexes due to the high energy demands of gestation and lactation, and because their feeding areas during the lactation period are constrained to a relatively small area in order to care for their pups [46]. In contrast, other age classes can travel longer distances to find prey. Like other colonies in the midriff area of the Gulf, G’s population has declined dramatically over the last two decades, likely because of decreased food availability [18]. Thus, adult female survival rates for this colony may be substantially lower than those observed at LI.

Other factors, like birth rates, may have contributed to the discrepancy between observed and predicted population trends for all colonies, particularly because our birth rate data derive from surrogate populations that are increasing (LI and SMI). However, we do not think birth rates are solely responsible for this difference because, although they influence population dynamics [21, 48], population growth of long lived mammals depends more on survival than reproduction [49].

Another possibility is that pup and juvenile survival rates were overestimated. However, this is unlikely because the expected bias would be lower rather than higher because of tag loss. Alternatively, pup and juvenile survival may have increased recently, and thus our estimated rates may overestimate those from the early part of the count interval. However, if declines continue on G, markedly lower adult survival rates are likely an important contributor to the mismatch. To help determine the reason for the current population decline at this colony, investigators should focus on estimating adult survival rates and cause-specific mortality. Factors that might contribute to lowered female adult survival include decreased prey availability, disease, and entanglement in fishing nets [18]. Finally, although we expect our counts of females on beaches to represents the least variable segment of the population, at any time of the year an unknown proportion are feeding at sea, dispersing, or simply not visible when counts are made and thus population size estimates based on these counts represent an index of unknown precision.

For SJ, the surrogate data yielded a relatively good fit between predicted and observed patterns. Nonetheless, our PVA model yields more pessimistic results than suggested by long-term counts due to the lowered pup and juvenile survival rates relative to the other colonies. We assume that current pup and juvenile survival rates are the same as they were in the past. However, SJ survival rates could have been higher than they are now, resulting in the relatively lower predicted growth. If the factors responsible for this reduced pup and juvenile survival also influence adult survival, our model would predict an even more pessimistic outlook for this colony in the future.

Surrogate data are typically thought of as data disjunct from the target population either spatially or taxonomically, but this study underscores that temporal disjunction is another form of surrogacy, and the same cautions should be applied when using data borrowed from other time intervals. Researchers should carefully consider whether current demographic data are effective for predicting the future or reconstructing the past of that same population. In this study, we used both current and past vital rates for LI to predict retrospective population dynamics, and the modest mismatches we observed likely arose from changing conditions through time relative to our fixed model parameters. More generally, these results support calls by other authors to use caution in interpreting PVA predictions for long time frames [50–52].

The intent of our analysis was to evaluate the effectiveness of using surrogate demographic data to predict the dynamics of other colonies of the same species in the same region, a common practice in wildlife studies. To do so, we modeled a scenario where researchers have little or no count data and must use surrogate vital rates to conduct a PVA. However, we recommend that when count data are available, integrated population models be used to improve model predictions, especially for models employing surrogate data. Integrated population models are a relatively novel quantitative tool that integrate demographic and count data into a single model. These models offer several advantages over the traditional approach (e.g. more precise parameter estimates), particularly for models with many parameters [53, 54].

Conservation plans for California sea lions in Mexico currently manage the entire Gulf as a unique demographic unit [18]. Our results suggest that a finer spatial scale should be considered in developing management strategies. Particular consideration should be given to G and other colonies in the midriff area of the Gulf that have declined over the last two decades and where > 90% of the population is concentrated [40]. Estimating survival rates for the complete life cycle of this species in other colonies of the Gulf and the Pacific coast of Mexico will allow ecologists to improve the reliability of management recommendations and ensure the persistence of these populations.

Our study provides valuable information about California sea lion life history patterns and a better understanding of the potential consequences of using surrogate vital rates from other species, other locations, or other time intervals to predict population trends. We recognize that for many imperiled species, vital rates are not available and immediate management actions must be taken. In those cases we recommend careful consideration of the potential for mismatches in surrogate data, and the use of surrogate data only when the populations appear to have similar population trends.

## Supporting Information

S1 File Field methods. Field methods for tag resighting and the preparation of data used in the survival analysis for young age classes at three colonies in the Gulf of California.
(DOCX)

S2 File Models examined for apparent survival rate (*φ*) (Table A) and resighting probability (*p*) (Table B). (DOCX)

S3 File Variable selection and behavioral sampling techniques. (DOCX)

S4 File Adjusted vital rates and model average resighting probabilities (p) for males and females. (DOCX)

S1 Table General characteristics of the study sites: San Jorge (SJ), Granito (G), and Los Islotes (LI). (DOCX)

S2 Table Field seasons, and number and percentage of male (M) and female (F) pups tagged at each sea lion colony relative to the total pup population. Pups were tagged in July every year except in 2008 when tagged pups from previous years were resighted.
(DOCX)
