# Supplementary material for: The Use of Surrogate Data in Demographic Population Viability Analysis: A Case Study of California Sea Lions
Source: PLoS One. 2015 Sep 28;10(9):e0139158. doi: 10.1371/journal.pone.0139158 (PMC4587556; doi:10.1371/journal.pone.0139158)
Supplement: S1 File — Field methods for tag resighting and the preparation of data used in the survival analysis for young age classes at three colonies in the Gulf of California. (DOCX) [file pone.0139158.s001.docx]

# Supporting Information

# S3 File. Field methods. Field methods for tag resighting and the preparation of data used in the survival analysis for young age classes at three colonies in the Gulf of California.

Here we present information omitted from the methods section due to space limitations.

**1. Tagging.**

We assumed pups were approximately one month old when they were tagged in July, since most births occur between the second and third weeks of June (June 15-21) and that the temporal distribution of births is similar at all rookeries [1]. Each pup was weighed to the nearest 0.25 kg using a 50 kg capacity spring scale.

**2. Tag resighting.**

We dedicated two 30-minute periods per day to resighting tagged sea lions at each site. A more significant amount of time was spent resighting tagged pups during the non-breeding season. The number of days invested on each colony was variable, although the number of hours/day dedicated to this activity was similar for all colonies (~8 hrs) (S2 Table). During the non-breeding season, we searched for tagged pups not only at the study sites, but also on other parts of the island from land and by boat. We expected to resight tagged pups and juveniles at other sites because they usually move to other areas after being weaned or following the breeding season [2].

We used 10 x 42 power binoculars and 20-60 x 60 power telescopes to search for tagged sea lions and read the codes from a distance (~50 m). We recorded the date, tag color, and tag code (whenever possible) for each resighting. When it was not possible to see or read the entire tag code, the resighting was registered, but not included in the survival analysis. We used the color of the tags as a second level of identification. If the sex of one individual based on its code did not match the sex indicated by the tag color, the identification was considered erroneous and the observation was discarded (0.06% of the resightings). Identification was considered positive when the status of at least one tag identifier was satisfactorily determined.

**3. Preparation of data used in the survival analysis.**

Multiple sightings of the same sea lion during one time period were coded as a single resighting event for that period. Resighting probabilities for colonies that were not visited during a particular period were fixed at 0 for all models. We classified individuals into two age classes: pups (< 1 year old) and juveniles (1-4 years old) [3]. Because the date and number of resighting trips varied for different years and colonies, we combined data from trips close in date (e.g., January 10, 2005 at LI and January 12, 2005 at G; S2 Table) to create a comparable resighting scheme for all colonies (Table A). We designated a single date (July 16) for the breeding season at each colony every year and then added in the various resighting trips performed through the years. The interval between each period was calculated by dividing the number of days between the midpoint of each resighting period by 365 to convert this number into a fraction of a year in order to estimate annual survival rates.

It was necessary to organize the data in this manner in order to fulfill the requirement of the MARK software that we input a common set of sampling occasions for each colony so that the time intervals between sampling occasions are the same for each colony. Had we instead used every sampling date as a sampling occasion, MARK would have created numerous sampling occasions with p=0 for colonies that weren’t sampled on that particular date, complicating the interpretation of the results. Moreover, the survival probability (φ) is likely to be 1 between two sampling occasions so close in time to one another. Thus, very little information is lost by combining sampling occasions into periods and treating the constituent occasions in a given period as a single one in order to reduce the number of sampling occasions.

**Table A.** Resighting periods and time intervals.

| Year | Period | Midpoint  (Month/Day) | Time interval* | Time  (Year/Period) |
| --- | --- | --- | --- | --- |
| 2004 | June-August | 07/16 |  |  |
| 2005 | January-February | 01/29 | 0.54 | 2004.54 |
|  | June-August | 07/16 | 0.46 | 2005 |
|  | September | 09/15 | 0.17 | 2005.17 |
| 2006 | January-February | 01/29 | 0.37 | 2005.54 |
|  | March-April | 03/30 | 0.16 | 2005.7 |
|  | June-August | 07/16 | 0.30 | 2006 |
|  | November-December | 11/30 | 0.38 | 2006.38 |
| 2007 | January-February | 01/29 | 0.16 | 2006.54 |
|  | March-April | 03/30 | 0.16 | 2006.7 |
|  | June-August | 07/16 | 0.30 | 2007 |
|  | September | 09/15 | 0.17 | 2007.17 |
|  | November-December | 11/30 | 0.21 | 2007.38 |
| 2008 | January-February | 01/29 | 0.16 | 2007.54 |
|  | March-April | 03/30 | 0.17 | 2007.7 |
|  | June-August | 07/16 | 0.30 | 2008 |

* # of days between midpoints/365 days.

**References**

1. García-Aguilar MC, Aurioles-Gamboa D. Breeding season of the California sea lion (*Zalophus californianus*) in the Gulf of California, Mexico. Aquatic Mammals. 2003; 29: 67–76.
2. Melin S, DeLong RL, Thomason JR, Vanblaricom GR. Attendance patterns of California sea lion (Z*alophus californianus*) females and pups during the non-breeding season at San Miguel Island. Marine Mammal Science. 2000; 16: 169-185.
3. Hernández-Camacho CJ, Aurioles-Gamboa D, Laake J, Gerber LR. Survival rates of the California sea lion*, Zalophus californianus*, in Mexico. Journal of Mammalogy. 2008; 89: 1059-1066.
