# Supplementary material for: The Use of Surrogate Data in Demographic Population Viability Analysis: A Case Study of California Sea Lions
Source: PLoS One. 2015 Sep 28;10(9):e0139158. doi: 10.1371/journal.pone.0139158 (PMC4587556; doi:10.1371/journal.pone.0139158)
Supplement: S2 File — (DOCX) [file pone.0139158.s002.docx]

# Supporting Information

**S4 File. Models examined for apparent survival rate (*φ*) (Table A) and resighting probability (*p*) (Table B).**

**Table A.** The 75 proposed models examined for apparent survival rate (φ). Age at resighting was grouped into two age classes (0, 1+). The effect of weight, density, and female aggression was limited to pups only. In some models, the sex and/or age class interaction was limited to juveniles only. Y = yes

| Model | Intercept | Age | Sex | Sex * Age | Colony | Time | Time * Age | Sex * Time *Age | Pup * Time | Pup * Weight | Pup * Density | Pup * Density^2^ | Pup * Aggression | Entanglement | Juvenile * Time |
| --- | --- | --- | --- | --- | --- | --- | --- | --- | --- | --- | --- | --- | --- | --- | --- |
| 1 | Y |  |  |  |  |  |  |  |  |  |  |  |  |  |  |
| 2 | Y | Y |  |  |  |  |  |  |  |  |  |  |  |  |  |
| 3 | Y |  | Y |  |  |  |  |  |  |  |  |  |  |  |  |
| 4 | Y | Y | Y |  |  |  |  |  |  |  |  |  |  |  |  |
| 5 | Y |  |  | Y |  |  |  |  |  |  |  |  |  |  |  |
| 6 | Y |  |  |  | Y |  |  |  |  |  |  |  |  |  |  |
| 7 | Y | Y |  |  | Y |  |  |  |  |  |  |  |  |  |  |
| 8 | Y |  | Y |  | Y |  |  |  |  |  |  |  |  |  |  |
| 9 | Y | Y | Y |  | Y |  |  |  |  |  |  |  |  |  |  |
| 10 | Y |  |  | Y | Y |  |  |  |  |  |  |  |  |  |  |
| 11 | Y |  |  |  | Y | Y |  |  |  |  |  |  |  |  |  |
| 12 | Y | Y |  |  | Y | Y |  |  |  |  |  |  |  |  |  |
| 13 | Y |  | Y |  | Y | Y |  |  |  |  |  |  |  |  |  |
| 14 | Y | Y | Y |  | Y | Y |  |  |  |  |  |  |  |  |  |
| 15 | Y |  |  | Y | Y | Y |  |  |  |  |  |  |  |  |  |
| 16 | Y |  |  |  |  | Y |  |  |  |  |  |  |  |  |  |
| 17 | Y | Y |  |  |  | Y |  |  |  |  |  |  |  |  |  |
| 18 | Y |  | Y |  |  | Y |  |  |  |  |  |  |  |  |  |
| 19 | Y | Y | Y |  |  | Y |  |  |  |  |  |  |  |  |  |
| 20 | Y |  |  | Y |  | Y |  |  |  |  |  |  |  |  |  |
| 21 | Y |  |  |  |  |  | Y |  |  |  |  |  |  |  |  |
| 22 | Y |  | Y |  |  |  | Y |  |  |  |  |  |  |  |  |
| 23 | Y |  |  |  |  |  |  | Y |  |  |  |  |  |  |  |
| 24 | Y |  |  | Y1+ |  |  |  |  |  |  |  |  |  |  |  |
| 25 | Y |  |  | Y1+ |  | Y |  |  |  |  |  |  |  |  |  |
| 26 | Y |  |  | Y1+ | Y |  |  |  |  |  |  |  |  |  |  |
| 27 | Y |  |  | Y1+ | Y | Y |  |  |  |  |  |  |  |  |  |
| 28 | Y |  |  | Y1+ |  |  |  |  | Y |  |  |  |  |  |  |

**Table A.** (cont.)

| Model | Intercept | Age | Sex | Sex * Age | Colony | Time | Time * Age | Sex * Time * Age | Pup * Time | Pup * Weight | Pup * Density | Pup * Density^2^ | Pup * Aggression | Entanglement | Juvenile * Time |
| --- | --- | --- | --- | --- | --- | --- | --- | --- | --- | --- | --- | --- | --- | --- | --- |
| 29 | Y |  |  | Y1+ | Y |  |  |  | Y |  |  |  |  |  |  |
| 30 | Y |  | Y |  | Y |  |  |  | Y |  |  |  |  |  |  |
| 31 | Y |  |  |  |  |  |  |  |  | Y |  |  |  |  |  |
| 32 | Y |  | Y |  |  |  |  |  |  | Y |  |  |  |  |  |
| 33 | Y |  |  | Y1+ |  |  |  |  |  | Y |  |  |  |  |  |
| 34 | Y |  |  |  |  |  |  |  |  |  | Y |  |  |  |  |
| 35 | Y |  | Y |  |  |  |  |  |  |  | Y |  |  |  |  |
| 36 | Y |  |  | Y1+ |  |  |  |  |  |  | Y |  |  |  |  |
| 37 | Y |  |  |  |  |  |  |  |  |  | Y | Y |  |  |  |
| 38 | Y |  | Y |  |  |  |  |  |  |  | Y | Y |  |  |  |
| 39 | Y |  |  | Y1+ |  |  |  |  |  |  | Y | Y |  |  |  |
| 40 | Y |  |  |  |  |  |  |  |  |  |  |  | Y |  |  |
| 41 | Y |  | Y |  |  |  |  |  |  |  |  |  | Y |  |  |
| 42 | Y |  |  | Y1+ |  |  |  |  |  |  |  |  | Y |  |  |
| 43 | Y |  |  |  |  |  |  |  |  |  | Y |  | Y |  |  |
| 44 | Y |  | Y |  |  |  |  |  |  |  | Y |  | Y |  |  |
| 45 | Y |  |  | Y1+ |  |  |  |  |  |  | Y |  | Y |  |  |
| 46 | Y |  |  |  |  |  |  |  |  |  | Y |  | Y | Y |  |
| 47 | Y |  | Y |  |  |  |  |  |  |  | Y |  | Y | Y |  |
| 48 | Y |  |  | Y1+ |  |  |  |  |  |  | Y |  | Y | Y |  |
| 49 | Y |  |  |  |  |  |  |  |  | Y | Y |  | Y |  |  |
| 50 | Y |  | Y |  |  |  |  |  |  | Y | Y |  | Y |  |  |
| 51 | Y |  |  | Y1+ |  |  |  |  |  | Y | Y |  | Y |  |  |
| 52 | Y |  |  |  |  |  |  |  |  | Y | Y |  |  |  |  |
| 53 | Y |  | Y |  |  |  |  |  |  | Y | Y |  |  |  |  |
| 54 | Y |  |  | Y1+ |  |  |  |  |  | Y | Y |  |  |  |  |
| 55 | Y |  |  |  |  |  |  |  |  | Y |  |  |  |  | Y |
| 56 | Y |  | Y |  |  |  |  |  |  | Y |  |  |  |  | Y |
| 57 | Y |  |  | Y1+ |  |  |  |  |  | Y |  |  |  |  | Y |
| 58 | Y |  |  |  |  |  |  |  |  |  | Y |  |  |  | Y |
| 59 | Y |  | Y |  |  |  |  |  |  |  | Y |  |  |  | Y |
| 60 | Y |  |  | Y1+ |  |  |  |  |  |  | Y |  |  |  | Y |
| 61 | Y |  |  |  |  |  |  |  |  |  | Y | Y |  |  | Y |

**STable A.** (cont.)

| Model | Intercept | Age | Sex | Sex * Age | Colony | Time | Time * Age | Sex * Time * Age | Pup *Time | Pup * Weight | Pup * Density | Pup * Density^2^ | Pup * Aggression | Entanglement | Juvenile * Time |
| --- | --- | --- | --- | --- | --- | --- | --- | --- | --- | --- | --- | --- | --- | --- | --- |
| 62 | Y |  | Y |  |  |  |  |  |  |  | Y | Y |  |  | Y |
| 63 | Y |  |  | Y1+ |  |  |  |  |  |  | Y | Y |  |  | Y |
| 64 | Y |  |  |  |  |  |  |  |  |  |  |  | Y |  | Y |
| 65 | Y |  | Y |  |  |  |  |  |  |  |  |  | Y |  | Y |
| 66 | Y |  |  | Y1+ |  |  |  |  |  |  |  |  | Y |  | Y |
| 67 | Y |  |  |  |  |  |  |  |  |  | Y |  | Y |  | Y |
| 68 | Y |  | Y |  |  |  |  |  |  |  | Y |  | Y |  | Y |
| 69 | Y |  |  | Y1+ |  |  |  |  |  |  | Y |  | Y |  | Y |
| 70 | Y |  |  |  |  |  |  |  |  | Y | Y |  | Y |  | Y |
| 71 | Y |  | Y |  |  |  |  |  |  | Y | Y |  | Y |  | Y |
| 72 | Y |  |  | Y1+ |  |  |  |  |  | Y | Y |  | Y |  | Y |
| 73 | Y |  |  |  |  |  |  |  |  | Y | Y |  |  |  | Y |
| 74 | Y |  | Y |  |  |  |  |  |  | Y | Y |  |  |  | Y |
| 75 | Y |  |  | Y1+ |  |  |  |  |  | Y | Y |  |  |  | Y |

**Table B.** The eight proposed models examined for resighting probability (*p*). Age at resighting was grouped into two age classes (0, 1+). In some models, the sex*age class interaction was limited to juveniles only. In some cases, the interaction between variables only includes a combination of factors that actually exist. For example, the colony/time interaction only created combinations of the colony during the time periods in which it was visited. Y = yes

| # Model | Intercept | Age | Sex | Sex * Age | Sex * Age * Colony * Time | Age * Colony * Time | Colony * Time | Breeding * Colony * Yearling |
| --- | --- | --- | --- | --- | --- | --- | --- | --- |
| 1 | Y |  |  |  | Y |  |  |  |
| 2 | Y |  | Y |  |  | Y |  |  |
| 3 | Y |  |  | Y |  |  | Y |  |
| 4 | Y |  |  | Y |  |  | Y | Y |
| 5 | Y | Y | Y |  |  |  | Y |  |
| 6 | Y | Y | Y |  |  |  | Y | Y |
| 7 | Y |  |  | Y |  |  | Y | Y |
| 8 | Y | Y |  |  |  |  | Y | Y |
