# Supplementary material for: The Use of Surrogate Data in Demographic Population Viability Analysis: A Case Study of California Sea Lions
Source: PLoS One. 2015 Sep 28;10(9):e0139158. doi: 10.1371/journal.pone.0139158 (PMC4587556; doi:10.1371/journal.pone.0139158)
Supplement: S3 File — (DOCX) [file pone.0139158.s003.docx]

# Supporting Information

**S5 File. Variable selection and behavioral sampling techniques.**

**1. Variable selection.**

We predicted that age and sex would affect survival and behavior (age-sex interaction) based on previous studies of this species [1, 2]. We also expected pup and juvenile survival to vary with time because young individuals are more vulnerable to interannual fluctuations in environmental conditions [1, 3, 4]. We expected survival to vary by colony because the colonies are located at different latitudes (i.e., exposed to different environmental conditions, available prey, etc.) and have distinct population trends [5-7].

The individual and site-specific covariates included in this analysis were pup weight at the time of tagging, female behavior (maternal attendance on land), female and pup density in territorial areas, and entanglement rates for all age classes. All covariates were measured in July, when most pups were tagged. By this time of year, most females have given birth and spend more time on land nursing newborns [8]. All covariates, except entanglement rates, were assumed to affect pups exclusively. A detailed explanation of how each of these variables was measured is presented in 2. Behavioral sampling (see below).

We expected pup survival, especially during the first months of life, to depend on the mother’s ability to obtain sufficient food and efficiently transfer energetic resources [9, 10]. Thus, pup weight reflects the level of maternal investment during the gestational and rearing periods. However, pup weight may also be influenced by individual differences in pup behavior and energy allocation (e.g., time invested playing vs. resting) [10, 11]. Therefore, we explored the effect of both maternal behavior (measured as the frequency of agonistic interactions) and pup weight on pup survival rates. We considered the frequency of female agonistic interactions to be an indicator of maternal investment because females involved in more aggressive encounters spend less time nursing their pups and are more likely to be separated from their pups [12, 13]. We didn’t consider the duration of nursing events because when we tested for differences in female behavior at different sites before including these variables in the survival analysis (see S3 File), only female aggression was significantly different between colonies, although this was not the case for different sites on the same colony. Female density also differed between colonies but not within sites (see [14]) and was not significantly related to female aggression (*r^2^* = 0.0006). Thus, this variable was included in the analysis.

We also predicted that incidental catch in fishing nets would affect pup and juvenile survival. The number of entangled sea lions recorded in colonies of the northern Gulf of California has increased over the last two decades, possibly contributing to the decline of sea lion populations in the area [15, 16].

Finally, we expected resighting probabilities to vary with age, sex, and colony. We also expected them to vary with time because of variation in resighting effort and fluctuations in environmental conditions. We accounted for differences in the methodology used to resight sea lions during the breeding and non-breeding season by creating the separate binary variable breeding (1 = breeding field trips and 0 = non-breeding field trips).

**2. Behavioral sampling.**

Behavioral observations were conducted during the first six days of each visit to SJ and G, and every day at LI (S2 Table). Behavioral observations at LI were scheduled at different times because this island is visited by tourists during the day.

We didn’t record and track the behavior of mothers of tagged pups because we were not able to identify individual females in the field. Thus, we recorded the behavior of the group of females present at each study site. The description of how each variable included in the survival analysis was measured is summarized in the table below.

We used repeated measures ANOVA to test for differences in the proportion of time females invested nursing their pups and the number of female aggression events per hour at each study site [17]. Assumptions of normality and homogeneity of variance were checked beforehand. All statistical analyses were computed using SAS System for Windows (9.1.3 SAS Institute, Inc., Cary, NC). Females from the same site were considered the same “subject” over the study period (July) since it was not possible to guarantee that each observation was independent. Female aggression was significantly different between colonies (*F* = 3.91, *p* = 0.04), but not between sites on the same colony (*F* = 1.07, *p* = 0.39). The proportion of time females invested nursing was not significantly different between colonies (*F* = 2.24 *p* = 0.15) or different sites on the same colony (*F* = 0.53, *p* = 0.67).

| Variable | | Description | Sampling description |
| --- | --- | --- | --- |
| Morphological | Pup weight | Most pups were weighed (tagged) in mid late July, when they were ca. one month old. | a) Pups from SJ in 2004 and pups from all colonies in 2006 were captured at the beginning of July and August so these individuals were younger/older and lighter/heavier than the rest of the pups sampled at the end of July. |
|  |  |  | b) To make this variable comparable between years, the weight of the pups captured earlier was “corrected” by multiplying the average daily growth rate* of males and females by the number of days elapsed between the day they were captured and the mean date of captures in the latter part of July. |
|  |  |  | c) The same calculation was applied to “correct” the weight of pups that were tagged in August 2006. In this case, weight gained between the mean capture date in July and the date of recapture in August was subtracted from the weight recorded in August. |
| Behavioral | Female agonistic interaction | Open mouth displays, vocalizations, grabbing, pushing or biting another female. | a) All interactions were recorded during one hour three times each day at each study site [14]. |
|  |  |  | b) Multiple same female-female pair interactions over a short period of time were considered a single event if they occurred within three minutes of one another. |
|  |  |  | c) Interactions initiated more than three minutes after the last bout were considered a new event. |
|  | Nursing | Female feeding pup. Time elapsed between pup attachment to the mother’s teat until withdrawal was considered a nursing event. | a) Five female-pup pairs were randomly selected at each study site and followed for one hour three times each day. |

| Variable | | Description | Sampling description |
| --- | --- | --- | --- |
| Behavioral | Nursing | Female feeding pup. Time elapsed between pup attachment to the mother’s teat until withdrawal was considered a nursing event. | b) Nursing data were not collected on the same pair more than once a day unless new pairs were not available at the beginning of the observation session. |
|  |  |  | c) Same female-pup pair nursing multiple times over a short period were considered a single event if they occurred within three minutes of one another, while events initiated more than three minutes after the last one were considered a new event. |
|  |  |  | d) When < five pairs were nursing at the beginning of the observational period, those pairs were followed for the entire period. New pairs were added as they started nursing until a sample of five pairs was obtained. |
| Others | Entanglement rate | Proportion of animals relative to the total population with remnants of fishing gear around their necks or heads. | a) Each colony was counted every year during July (S2 Table). Counts were conducted around noon by circumnavigating the island in a boat with an outboard engine. |
|  |  |  | b) Sea lions were classified by age and sex [18]. |
|  |  |  | c) A correction factor of 50% for pups and 54% for females was applied following [19]. |
|  |  |  | d) Annual entanglement rates for each colony were calculated by dividing the total number of entangled sea lions of each category by the total number of sea lions of the same category observed every year. |
|  | Density | Average number of females and pups observed at the study site in July divided by the area of the site. | a) Counts were performed at each study site three times a day every day (six days in July). |
|  |  |  | b) The area of each site was estimated by building polygons in ArcGIS using the GPS coordinates of each site’s perimeter (land and water) [14]. |

* Daily average growth rate of individuals captured in July was calculated as the difference in weight between the first and last capture divided by the number of days elapsed between the two dates. Growth rate: males (2004 SJ = 0.14 SE 0.027; 2006 SJ = 0.09 SE 0.017, G = 0.11 SE 0.013, LI = 0.18 SE 0.017) and females (2004: SJ = 0.095 SE 0.016; 2006: SJ = 0.09 SE 0.025, G = 0.08 SE 0.015, LI = 0.12 SE 0.025).

**References**

1. Hernández-Camacho CJ, Aurioles-Gamboa D, Laake J, Gerber LR. Survival rates of the California sea lion*, Zalophus californianus*, in Mexico. Journal of Mammalogy. 2008; 89: 1059-1066.
2. Laake J, DeLong R, Melin S. Age- and sex-specific survivorship of California sea lions. Sea Lions of the World. Conservation and Research in the 21^st^ Century. 22^nd^ Wakefield Fisheries Symposium. September 30-October 3 Anchorage, Alaska, USA. 2004.
3. Beauplet G, Barbraud C, Chambellant M, Guinet C. Interannual variation in the post-weaning and juvenile survival of Subantarctic fur seals: influence of pup sex, growth rate and oceanographic conditions. Journal of Animal Ecology. 2005; 74: 1160–1172.
4. Clutton-Brock TH, Illius A, Wilson K, Grenfell TB, Maccoll A, Albon SD. Stability and instability in ungulate populations: an empirical analysis. American Naturalist. 1997; 149: 195-219.
5. Lluch-Cota SE, Aragón-Noriega EA, Arreguín-Sánchez F, Aurioles-Gamboa D, Bautista-Romero J, Brusca RC. et al. The Gulf of California: Review of ecosystem status and sustainability challenges. Progress in Oceanography. 2007; 73:1–26.
6. Porras-Peters H, Aurioles-Gamboa D, Cruz-Escalona VH, Koch PL. Trophic level and overlap of sea lions (*Zalophus californianus*) in the Gulf of California, México. Marine Mammal Science. 2008; 24:554-576.
7. Ward EJ, Chrirakkal H, González-Suárez M, Aurioles-Gamboa D, Holmes EE, Gerber L. Inferring spatial structure from time-series data: using multivariate state-space models to detect metapopulation structure of California sea lions in the Gulf of California, Mexico. Journal of Applied Ecology. 2010; 47:47-56.
8. García-Aguilar MC, Aurioles-Gamboa D. Breeding season of the California sea lion (*Zalophus californianus*) in the Gulf of California, Mexico. Aquatic Mammals. 2003; 29: 67–76.
9. Bowen WD. Behavioral ecology of pinniped neonates. In Renouf D. editors. The behavior of pinnipeds*.* Cambridge University Press, New York, USA. 1991. pp 66-124.
10. Ono KA, Boness DJ. Sexual dimorphism in sea lion pups: differential maternal investment, or sex-specific differences in energy allocation? Behavioral Ecology and Sociobiology. 1996; 38:31-41.
11. Hall AJ, McConnell BJ, Barker RJ. Factors affecting first-year survival in grey seals and their implications for life history strategy. Journal of Animal Ecology. 2001; 70: 138-149.
12. Cassini MH, Fernández-Juricic E. Cost and benefits of joining South American sea lion breeding groups: the assumptions of a model of female breeding dispersion. Canadian Journal of Zoology. 2003; 81: 1154-1160.
13. Harcourt R. Maternal aggression in the South American fur seal in Peru. Canadian Journal of Zoology. 1991; 70: 320-325.
14. Young KJ, González-Suárez M, Gerber LR. Determinants of agonistic interactions in California sea lions. Behaviour. 2008; 145:1597-1810.
15. Underwood JG, Hernández-Camacho CJ, Aurioles-Gamboa D, Gerber LR. Estimating sustainable bycatch rates for California sea lion populations in the Gulf of California. Conservation Biology. 2008; 22:701-710.
16. Zavala-González A, Mellink E. Entanglement of California sea lions, *Zalophus californianus californianus*, in fishing gear in the central-northern part of the Gulf of California. Fishery Bulletin. 1997; 95:180-184.
17. Kutner HM, Nachtsheim CJ, Neter J, Li W. Applied linear statistical models. McGraw-Hill, New York, USA; 2005.
18. Le Boeuf BJ, Aurioles-Gamboa D, Condit R, Fox C, Gisiner R, Romero R, et al. Size and distribution of the California sea lion population in Mexico. Proceedings of the California Academy of Sciences. 1983; 43: 77–85.
19. Szteren D, Aurioles-Gamboa D, Gerber L. Population status and trends of the California sea lion (*Zalophus californianus*) in the Gulf of California, Mexico. In Trites AW, Atkinson SK, DeMaster DP, Fritz LW, Gelatt ST, Rea LD, Wynne KM, editors. Sea lions of the world. Alaska Sea Grant College Program, University of Alaska, Fairbanks, Alaska; 2006. pp. 369-384.
