# Supplementary material for: The Use of Surrogate Data in Demographic Population Viability Analysis: A Case Study of California Sea Lions
Source: PLoS One. 2015 Sep 28;10(9):e0139158. doi: 10.1371/journal.pone.0139158 (PMC4587556; doi:10.1371/journal.pone.0139158)
Supplement: S4 File — (DOCX) [file pone.0139158.s004.docx]

# Supporting Information

**S6 File. Adjusted vital rates and model average resighting probabilities (p) for males and females.**

**Table A.** Adjusted vital rates from the top model φ (14) for survival and from birth rates (B_i_) estimated by [1, 2]. The additive model for survival was selected because annual time variability in pup and juvenile survival was included. Survival rates for adults come from a single population (LI) and there was no annual variability in survival rates [3]. The age classes used are: pups (< 1 year old), juveniles, prime age adults, and old adults. Prime age and old adult survival rates come from [3].

| Survival Rates | Males | | Females | |
| --- | --- | --- | --- | --- |
| Age class/ Colony | φ | Process variance | φ | Process  variance |
| Pups |  |  |  |  |
| SJ | 0.949 | 0.005 | 0.831 | 0.032 |
| G | 0.975 | 0.000 | 0.942 | 0.001 |
| LI | 0.992 | 0.000 | 0.942 | 0.006 |
|  |  |  |  |  |
| Juveniles |  |  |  |  |
| SJ | 0.748 | 0.097 | 0.606 | 0.165 |
| G | 0.971 | 0.005 | 0.874 | 0.037 |
| LI | 0.882 | 0.033 | 0.741 | 0.098 |
|  |  |  |  |  |
| Prime age Adults | 0.904 | 0.000 | 0.970 | 0.000 |
|  |  |  |  |  |
|  |  |  |  |  |
| Old Adults | 0.746 | 0.000 | 0.909 | 0.000 |

| Birth rates for females |  |  |  |
| --- | --- | --- | --- |
|  | Age class (i) | B_i_ | Process variance |
|  | 5 | 0.593 | 0.017 |
|  | 6-9 | 0.813 | 0.004 |
|  | 10-12 | 0.818 | 0.002 |
|  | 13-15 | 0.679 | 0.040 |
|  | 16-18 | 0.673 | 0.012 |
|  | 19-21 | 0.647 | 0.032 |
|  | 22-25 | 0.001 | 0.000 |

**Table B.** Model average resighting probabilities (*p*) for males and females. Pups and juveniles were resighted at different ages depending on the colony. The SE is shown in parentheses.

| Island | Time | Age | Males | | Females | |
| --- | --- | --- | --- | --- | --- | --- |
|  |  |  | Pups | Juveniles | Pups | Juveniles |
| SJ | 2004.54 | 0.54 | 0.032 (0.032) |  | 0.111 (0.061) |  |
|  |  |  |  |  |  |  |
|  |  |  |  |  |  |  |
|  | 2006.54 | 0.54 | 0.374 (0.075) |  | 0.398 (0.078) |  |
|  |  |  |  |  |  |  |
|  |  |  |  |  |  |  |
|  | 2007.54 | 0.54 | 0.255 (0.092) |  | 0.292 (0.095) |  |
|  |  |  |  |  |  |  |
|  |  |  |  |  |  |  |
|  | 2006.38 | 0.38 | 0.390 (0.074) |  | 0.580 (0.078) |  |
|  |  |  |  |  |  |  |
|  |  |  |  |  |  |  |
|  | 2005.17 | 0.17 | 0.468 (0.072) |  | 0.643 (0.079) |  |
|  |  |  |  |  |  |  |
|  |  |  |  |  |  |  |
|  | 2007.17 | 0.17 | 0.930 (0.054) |  | 0.753 (0.091) |  |
|  |  |  |  |  |  |  |
|  |  |  |  |  |  |  |
|  | 2005.7 | 0.7 | 0.360 (0.072) |  | 0.513 |  |
|  |  |  |  |  |  |  |
|  |  |  |  |  | (0.092) |  |
|  | 2005 | 1 |  | 0.069 (0.047) |  | 0.172  (0.082) |
|  | 2006 | 1 |  | 0.108 (0.046) |  | 0.476  (0.095) |
|  | 2007 | 1 |  | 0.046 (0.032) |  | 0.236  (0.074) |
|  | 2006 | 2 |  | 0.000 (0.000) |  | 0.116  (0.079) |

**Table B.** (cont.)

| Island | Time | Age | Males | | Females | |
| --- | --- | --- | --- | --- | --- | --- |
|  |  |  | Pups | Juveniles | Pups | Juveniles |
| SJ | 2007 | 2 |  | 0.015 (0.015) |  | 0.15  (0.061) |
|  |  |  |  |  |  |  |
|  |  | 3 |  |  |  |  |
|  | 2008 | 2 |  | 0.000 (0.000) |  | 0.024  (0.027) |
|  |  |  |  |  |  |  |
|  |  | 3 |  |  |  |  |
|  |  |  |  |  |  |  |
|  |  | 4 |  |  |  |  |
|  | 2006.38 | 2.38 |  | 0.332 (0.064) |  | 0.285  (0.071) |
|  |  |  |  |  |  |  |
|  |  | 1.38 |  |  |  |  |
|  | 2008.38 | 2.38 |  | 0.021 (0.016) |  | 0.18  (0.079) |
|  |  |  |  |  |  |  |
|  |  | 1.38 |  |  |  |  |
|  |  |  |  |  |  |  |
|  |  | 3.38 |  |  |  |  |
|  |  |  |  |  |  |  |
|  |  | 4.38 |  |  |  |  |
|  | 2006.54 | 2.54 |  | 0.088 (0.035) |  | 0.227  (0.067) |
|  |  |  |  |  |  |  |
|  |  | 1.54 |  |  |  |  |
|  | 2007.54 | 2.54 |  | 0.285 (0.082) |  | 0.411  (0.114) |
|  |  |  |  |  |  |  |
|  |  | 3.54 |  |  |  |  |
|  |  |  |  |  |  |  |
|  |  | 1.54 |  |  |  |  |
| G | 2004.54 | 0.54 | 0.325 (0.061) |  | 0.589 (0.085) |  |
|  |  |  |  |  |  |  |
|  |  |  |  |  |  |  |
|  | 2006.38 | 0.38 | 0.232 (0.053) |  | 0.290 (0.074) |  |
|  |  |  |  |  |  |  |
|  |  |  |  |  |  |  |
|  | 2006.7 | 0.7 | 0.592 (0.062) |  | 0.402 (0.083) |  |
|  |  |  |  |  |  |  |
|  |  |  |  |  |  |  |

**Table B.** (cont.)

| Island | Time | Age | Males | | Females | |
| --- | --- | --- | --- | --- | --- | --- |
|  |  |  | Pups | Juveniles | Pups | Juveniles |
| G | 2007.38 | 0.38 | 0.787 (0.086) |  | 0.738 (0.089) |  |
|  |  |  |  |  |  |  |
|  |  |  |  |  |  |  |
|  | 2005 | 1 |  | 0.000 (0.000) |  | 0.026  (0.026) |
|  | 2005.7 | 1.7 |  | 0.230 (0.057) |  | 0.397  (0.091) |
|  | 2006 | 2 |  | 0.000 (0.000) |  | 0.000  (0.000) |
|  | 2006.38 | 2.38 |  | 0.128 (0.038) |  | 0.14  (0.048) |
|  |  |  |  |  |  |  |
|  |  | 1.38 |  |  |  |  |
|  | 2006.7 | 2.7 |  | 0.168 (0.044) |  | 0.126  (0.047) |
|  |  |  |  |  |  |  |
|  |  | 1.7 |  |  |  |  |
|  | 2007 | 3 |  | 0.013 (0.013) |  | 0.000  (0.000) |
|  |  |  |  |  |  |  |
|  |  | 2 |  |  |  |  |
|  | 2007.38 | 3.38 |  | 0.200 (0.038) |  | 0.285  (0.062) |
|  |  |  |  |  |  |  |
|  |  | 2.38 |  |  |  |  |
|  |  |  |  |  |  |  |
|  |  | 1.38 |  |  |  |  |
|  | 2007.71 | 3.71 |  | 0.208 (0.044) |  | 0.219  (0.064) |
|  |  |  |  |  |  |  |
|  |  | 2.71 |  |  |  |  |
|  |  | 1.71 |  |  |  |  |
|  | 2008.38 | 4.38 |  | 0.047 (0.019) |  | 0.031  (0.019) |
|  |  |  |  |  |  |  |
|  |  | 3.38 |  |  |  |  |
|  |  |  |  |  |  |  |
|  |  | 2.38 |  |  |  |  |
|  |  | 1.38 |  |  |  |  |

**Table B.** (cont.)

| Island | Time | Age | Males | | Females | |
| --- | --- | --- | --- | --- | --- | --- |
|  |  |  | Pups | Juveniles | Pups | Juveniles |
| G | 2006 | 1 |  | 0.128 (0.069) |  | 0.040  (0.040) |
|  | 2007 | 1 |  | 0.250 (0.055) |  | 0.082  (0.046) |
| LI | 2004.54 | 0.54 | 0.078 (0.038) |  | 0.284 (0.075) |  |
|  |  |  |  |  |  |  |
|  |  |  |  |  |  |  |
|  | 2005.54 | 0.54 | 0.255 (0.070) |  | 0.258 (0.079) |  |
|  |  |  |  |  |  |  |
|  |  |  |  |  |  |  |
|  | 2006.54 | 0.54 | 0.137 (0.057) |  | 0.341 (0.105) |  |
|  |  |  |  |  |  |  |
|  |  |  |  |  |  |  |
|  | 2007.54 | 0.54 | 0.537 (0.095) |  | 0.548 (0.107) |  |
|  |  |  |  |  |  |  |
|  |  |  |  |  |  |  |
|  | 2005 | 1 |  | 0.243 (0.063) |  | 0.393  (0.091) |
|  | 2005.54 | 1.54 |  | 0.211 |  | 0.092 |
|  |  |  |  | (0.062) |  | (0.052) |
|  |  |  |  |  |  |  |
|  | 2006.54 | 2.54 |  | 0.258  (0.053) |  | 0.018  (0.018) |
|  |  |  |  |  |  |  |
|  |  |  |  |  |  |  |
|  |  |  |  |  |  |  |
|  |  | 1.54 |  |  |  |  |
|  | 2007 | 3 |  | 0.126 (0.040) |  | 0.282  (0.074) |
|  |  |  |  |  |  |  |
|  |  | 2 |  |  |  |  |
|  | 2007.54 | 3.54 |  | 0.228 (0.054) |  | 0.309  (0.083) |
|  |  |  |  |  |  |  |
|  |  | 2.54 |  |  |  |  |
|  |  |  |  |  |  |  |
|  |  | 1.54 |  |  |  |  |

**Table B.** (cont.)

| Island | Time | Age | Males | | Females | |
| --- | --- | --- | --- | --- | --- | --- |
|  |  |  | Pups | Juveniles | Pups | Juveniles |
| LI | 2008 | 4 |  | 0.219 (0.069) |  | 0.193  (0.085) |
|  |  |  |  |  |  |  |
|  |  | 3 |  |  |  |  |
|  |  |  |  |  |  |  |
|  |  | 2 |  |  |  |  |
|  | 2006 | 1 |  | 0.284 (0.073) |  | 0.268  (0.082) |

Pups and juveniles from SJ had the lowest survival rates, while those from G had the highest. Females had lower survival rates than males in all age classes on all colonies.

The high pup and juvenile survival rates reported here fall within the range reported for LI colony in the early 1980s [3], although female juvenile survival rates have declined slightly.

Resighting probability varied with time, age, sex, and colony. Resighting probability values were low compared to those for individuals of the same age and sex classes observed in a previous study of branded sea lions from LI [3]. This difference is probably due to difficulties in reading flipper tags compared to reading brands when animals congregate on land. In addition, when animals are submerged in water it is more difficult to read tags than to identify branding marks. Resighting probabilities were similar for males and females from LI, as was the case in an earlier study [3].

Variability in pup resighting probabilities for different colonies may be due to differences in substrate (sandy beaches at G vs. platform rocks at LI), animal density, animal sensitivity to the presence of humans, and resighting conditions (e.g., distance between observer and animal, number of observation areas on each colony) [4]. For juveniles, differences between colonies are due to: 1) the LI colony being smaller (area and population size), making it more likely that juveniles be resighted; 2) juvenile sea lions living in the northern Gulf of California, where 76% of breeding colonies are located, exploring other nearby colonies, while the counterparts at the LI colony are relatively isolated.

Resighting probabilities were lower for juveniles than for pups. Pup resighting rates were lower for LI and higher for SJ and G, while juvenile resighting rates showed the opposite trend. Overall, pup and juvenile males had a lower resighting rate than females. Resighting rates varied across years in all age and sex classes and colonies.

Survival estimates may be biased due to lost tags and the rate of tag loss may vary between colonies due to differences in terrain (rocky versus sandy substrate), population density (the higher the density, the greater the probability that physical contact with conspecifics will dislodge tags), behavior (very active versus less active individuals), and other factors [4]. However, we consider the rate of tag loss and the bias in the survival estimation to be negligible during the study period for the following reasons: 1) we observed only one individual with a scar left by a tag on one of its flippers during the study period, 2) the rate of tag loss on sea lions at SMI was estimated to be <10% for individuals <3 years old (J. L., unpublished data), and 3) LI survival estimates were higher than those obtained from a mark-recapture analysis based on hot branded sea lions from the same colony [3]. If the rate of tag loss were high, we would expect to have lower survival estimates.

**References**

1. Hernández-Camacho CJ, Aurioles-Gamboa D, Gerber LR. Age-specific birth rates of California sea lions (*Zalophus californianus*) in the Gulf of California, Mexico*.* Marine Mammal Science. 2008; 24:664-676.
2. Melin RS, Laake JL, DeLong RL, Siniff DB. Age-specific recruitment and natality of California sea lions at San Miguel Island, California. Marine Mammal Science. 2011; 28:751-776
3. Hernández-Camacho CJ, Aurioles-Gamboa D, Laake J, Gerber LR. Survival rates of the California sea lion*, Zalophus californianus*, in Mexico. Journal of Mammalogy. 2008; 89:1059-1066.
4. Bradshaw CJA, Barker RJ, Lloyd SD. Modeling tag loss in New Zealand fur seal pups. Journal of Agricultural, Biological, and Environmental Statistics. 2000; 5**:**475-785.
