# Supplementary material for: The Use of Surrogate Data in Demographic Population Viability Analysis: A Case Study of California Sea Lions
Source: PLoS One. 2015 Sep 28;10(9):e0139158. doi: 10.1371/journal.pone.0139158 (PMC4587556; doi:10.1371/journal.pone.0139158)
Supplement: S1 Table — (DOCX) [file pone.0139158.s005.docx]

# Supporting Information

**S1 Table.** General characteristics of the study sites: San Jorge (SJ), Granito (G), and Los Islotes (LI).

| Colony | N^a^ | Growth tendency^b^ | Entanglement rate^c^ | | | Tourism^d^ |
| --- | --- | --- | --- | --- | --- | --- |
|  |  |  | 1992 2004-2007 | | |  |
|  |  |  |  |  |  |  |
| SJ | 2,515 | Declining | 0.7 |  | 1.55 | Medium |
| G | 907 | Declining | 0.6 |  | 0.2 | Low |
| LI | 567 | Increasing | 10 |  | 0.83 | High |

^a^Population size in 2008 (L.G. and D. A.-G., unpublished data).

^b^According to [1] (SJ: 1985-2004, G:1991-2004, LI:1993-2004)

^c^Entanglement rate (Number of entangled sea lions registered while counting individuals present at a particular colony divided by the total population from that year X 100) (1992 [2], D. A.-G., unpublished data; 2004-2007 L. G., unpublished data).

^d^Frequency of human exposure (Number of visitors per hour during the breeding season) [3, 4].

**References**

1. Szteren D, Aurioles-Gamboa D, Gerber L. Population status and trends of the California sea lion (*Zalophus californianus*) in the Gulf of California, Mexico. In Trites AW, Atkinson SK, DeMaster DP, Fritz LW, Gelatt ST, Rea LD, Wynne KM, editors. Sea lions of the world. Alaska Sea Grant College Program, University of Alaska, Fairbanks, Alaska; 2006. pp. 369-384.
2. Zavala-González A, Mellink E. Entanglement of California sea lions, *Zalophus californianus californianus*, in fishing gear in the central-northern part of the Gulf of California. Fishery Bulletin. 1997; 95: 180-184.
3. French SS, González-Suárez M, Young JK, Durham S, Gerber LR. Human disturbance influences reproductive success and growth rate in California sea lions (*Zalophus californianus*). PLoS ONE. 2011; 6: e17686.
4. Labrada-Martagón V, Aurioles-Gamboa D, Martínez-Díaz SF. Natural and human disturbance in a rookery of the California sea lion (*Zalophus californianus*) in the Gulf of California, Mexico. Latin American Journal of Aquatic Mammals. 2005; 4:175–185.
