# Supplementary material for: The Use of Surrogate Data in Demographic Population Viability Analysis: A Case Study of California Sea Lions
Source: PLoS One. 2015 Sep 28;10(9):e0139158. doi: 10.1371/journal.pone.0139158 (PMC4587556; doi:10.1371/journal.pone.0139158)
Supplement: S2 Table — Pups were tagged in July every year except in 2008 when tagged pups from previous years were resighted. (DOCX) [file pone.0139158.s006.docx]

# Supporting Information

**S2 Table.** Field seasons, and number and percentage of male (M) and female (F) pups tagged at each sea lion colony relative to the total pup population. Pups were tagged in July every year except in 2008 when tagged pups from previous years were resighted.

| Island / Season | 2004 | | 2005 | | 2006 | | 2007 | | 2008 | Total |
| --- | --- | --- | --- | --- | --- | --- | --- | --- | --- | --- |
|  | Date | # Pups | Date | # Pups | Date | # Pups | Date | # Pups | Date |  |
|  |  | M/F |  | M/F |  | M/F |  | M/F |  |  |
|  |  | (%) |  | (%) |  | (%) |  | (%)` |  |  |
| San Jorge | | | | | | | | | | |
| B | 07/10-17 | 34/33 |  | 50/38 | 07/09-14 | 48/56 |  | 25/37 |  | 157/164 |
|  |  | (3.2) | 07/15-20 | (8.5) | 08/06-11 | (4.3) | 07/22-27 | (3.7) |  |  |
|  |  |  |  |  |  |  |  |  |  |  |
| NB |  |  | 02/05 |  | 04/21-23 |  | 02/12-14 |  | 02/23-24 |  |
|  |  |  | 09/24-25 |  | 11/10-13 |  | 09/23-24 |  |  |  |
| Granito | | | | | | | | | | |
| B | 07/03 | 59/41 | 07/15-20 | 24/26 | 07/09-14 | 66/40 |  | 26/31 |  | 175/138 |
|  | 07/21-26 | (29.8) |  | (18.8) | 08/06-11 | (23.3) | 07/22-27 | (35.8) |  |  |
|  |  |  |  |  |  |  |  |  |  |  |
| NB |  |  | 01/12-14 |  | 03/13-16 |  | 03/13-15 |  | 03/11-13 |  |
|  |  |  |  |  | 12/10-11 |  | 11/09-12 |  |  |  |
| Los Islotes | | | | | | | | | | |
| B | 07/01-07 | 53/43 | 07/21-28 | 40/32 | 07/08-14 | 37/21 |  | 31/28 |  | 161/124 |
|  | 07/21-27 | (85.3) |  | (76.2) | 08/04-10 | (75.8) | 07/22-28 | (70.2) |  |  |
|  |  |  |  |  |  |  |  |  |  |  |
| NB |  |  | 01/10-12 |  | 01/11-12 |  | 01/09-10 |  | 01/04-05 |  |

# B = Breeding season, NB = Non breeding season
